# Thyrotoxicosis with absence of clinical features of acromegaly in a TSH- and GH-secreting, invasive pituitary macroadenoma

**DOI:** 10.1530/EDM-14-0070

**Published:** 2015-01-01

**Authors:** Philip C Johnston, Amir H Hamrahian, Richard A Prayson, Laurence Kennedy, Robert J Weil

**Affiliations:** Department of Endocrinology Diabetes and Metabolism, Cleveland Clinic Foundation, 9500 Euclid Avenue Desk F20, Cleveland, Ohio, 44195, USA; 1 Patholgy and Laboratory Medicine Institute, Rose Ella Burkhardt Brain Tumor and Neuro-Oncology Center, Cleveland Clinic, Cleveland, Ohio, 44195, USA; 2 Department of Neurosurgery and the Neurological Institute, Rose Ella Burkhardt Brain Tumor and Neuro-Oncology Center, Cleveland Clinic, Cleveland, Ohio, 44195, USA

## Abstract

A 54-year-old woman presented with bi-temporal hemianopia, palpitations, and diaphoresis. An invasive pituitary macroadenoma was discovered. The patient had biochemical evidence of secondary hyperthyroidism and GH excess; however, she did not appear to be acromegalic. Surgical removal of the pituitary mass revealed a plurihormonal TSH/GH co-secreting pituitary adenoma. TSH-secreting adenomas can co-secrete other hormones including GH, prolactin, and gonadotropins; conversely, co-secretion of TSH from a pituitary adenoma in acromegaly is infrequent.

**Learning points:**

- This case highlights an unusual patient with a rare TSH/GH co-secreting pituitary adenoma with absence of the clinical features of acromegaly.
- Plurihormonality does not always translate into the clinical features of hormonal excess.
- There appears to be a clinical and immunohistochemical spectrum present in plurihormonal tumors.

## Background

Thyrotropin (TSH)-secreting pituitary adenomas are rare and account for 1% or fewer of all pituitary adenomas. Approximately one-third of TSH-secreting pituitary adenomas co-secrete other hormones, the most common of which is growth hormone (GH) (16%) followed by prolactin (11%) and gonadotropins (1%). Conversely, in GH-producing pituitary adenomas, 13% have been shown to demonstrate immunopositivity to TSH (1) (2). Plurihormonal pituitary tumors are either morphologically monomorphous (single cells producing different hormones) or plurimorphous (different cells producing different hormones). Plurihormonal pituitary tumors seem to predict a higher risk of tumor recurrence, in comparison to tumors that secrete only one hormone; therefore, careful follow-up of this population is essential (3) (4). This report describes a rare case of a TSH/GH co-secreting pituitary adenoma with the absence of features of acromegaly.

## Case presentation

A 54-year-old woman was noted on a routine visit to her ophthalmologist to have bi-temporal hemianopia. She had reported difficulty with reading, but had not noted peripheral vision loss. Her medical history included atrial fibrillation, hypertension, cardiomyopathy, and sleep apnea. On review of symptoms, she reported hirsutism, feeling ‘hot’, palpitations, and diaphoresis. Physical examination revealed an obese woman with a BMI of 44, hirsutism, a moon-shaped face, and supraclavicular fullness. She had no goiter or dysthyroid eye disease. She had no overt features of acromegaly such as coarse facial features or broad fingers.

## Investigation

Bi-temporal hemianopia was present, and magnetic resonance imaging (MRI) of the sella revealed a large pituitary macroadenoma measuring 2.3 cm and left cavernous sinus invasion, with suprasellar extension compressing the optic chiasm, principally to the left of midline. Initial laboratory testing revealed central hyperthyroidism: thyroxine (T_4_) 11.4 (5.0–11.0 μg/dl), free thyroxine (fT_4_) 2.1 (0.7–1.8 ng/dl), TSH 2.8 (0.4–5.5 μU/ml), and tri-iodothyronine (T_3_) 185 (94–170 ng/dl), in addition to hypersomatotropism: insulin-like growth factor 1 (IGF1) 747 (87–267 ng/ml) and GH 1.58 ng/ml (Table 1).

**Table 1:** Pituitary hormonal profile at presentation

**Test**	**Value**	**Normal range**
fT_4_	2.1	0.7–1.8 ng/dl
TSH	2.8	0.4–5.5 μU/ml
T_3_	185	94–170 ng/dl
Prolactin	1.5	2.1–17.4 ng/ml
GH (basal)	1.58	0.01–0.97 ng/ml
IGF1	747	87–267 ng/ml
Cortisol^a^	6.6, 24.1, 15.8	>18 μg/dl
ACTH (am)	16	8–42 pg/ml
24-h urine cortisol	16.3	<45 μg/day

T_4_, total thyroxine; fT_4_, free thyroxine; TSH, thyroid-stimulating hormone; T_3_, tri-iodothyronine; GH, growth hormone; IGF1, insulin-like growth factor 1; ACTH, adrenocorticotropic hormone.

a Short synacthen test: serum cortisol at baseline, +30, and +60 min respectively after administration of 250 μg cosyntropin.

## Treatment

**Figure 1:**
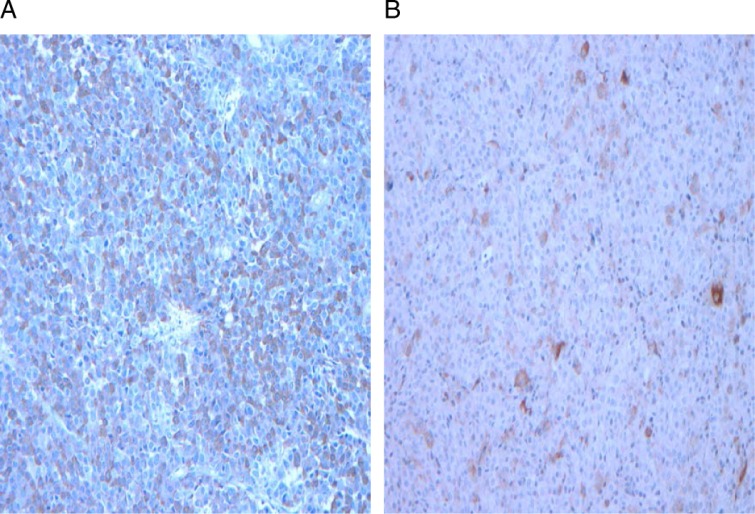
Immunohistochemical staining results. (A) The adenoma shows diffuse staining for antibody againt growth hormone (GH). (B) There is sparse and much weaker staining for antibody directed against TSH. Positively staining cells are brown.

In the context of the initial biochemistry indicating a plurihormonal TSH/GH co-secreting pituitary adenoma, pre-operative treatment with methimazole and octreotide was initiated. She proceeded to transsphenoidal resection of the pituitary macroadenoma 2 weeks later, following a reduction in her diaphoresis, hot flashes, and palpitations. At surgery, a sellar and suprasellar tumor was removed; the diaphragma sella was identified, ballotted down into the sellar region with the pulsations of the cerebrospinal fluid when the tumor was removed, and there was no dehiscence of the diaphragm or transgression of it by tumor identified upon careful inspection after tumor resection; it was believed that a gross total resection was effected. The normal pituitary gland, which was displaced superiorly and to the right, was thin and atretic. Immunohistochemical staining of the adenoma with antibodies to adrenocorticotropic hormone (ACTH), GH, follicle-stimulating hormone, luteinizing hormone, prolactin (PRL), and TSH was performed. Immunoreactivity with antibody directed against GH was strong and diffuse, while that of TSH was exceptionally rare and scattered throughout the adenoma (Fig. 1A, GH and B, TSH, show representative staining; positive cells are brown). The adenoma did not stain with any of the other above-mentioned antibodies. Methimazole and octreotide were stopped on the day of surgery. Post-operatively, the symptoms and signs of hyperthyroidism resolved rapidly.

## Outcome and follow-up

**Figure 2:**
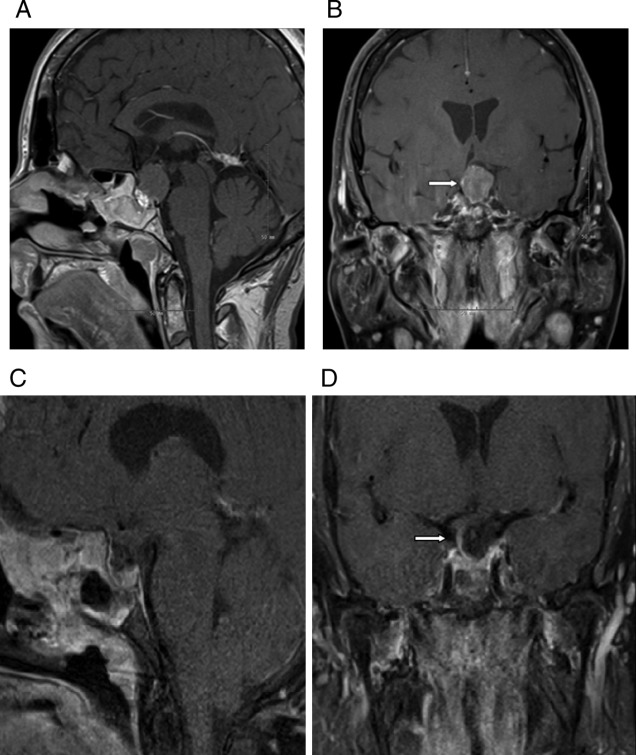
Magentic resonance imaging results. (A and B) T1-weighted, post-gadolinium coronal (A) and sagittal (B) images demonstrating a large pituitary macroadenoma (arrow) present on sellar imaging 6 weeks after the initial surgery; a fat graft (with very high signal intensity) lies in the floor of the sella just anteroinferior to the compressed normal gland (best observed on the coronal view, A). The normal gland, lying in the floor of the enlarged sella, enhances avidly and is compressed by the suprasellar tumor, as best appreciated on the sagittal view. The tumor was suprasellar, above the diaphragm as observed at surgery, and compressed the diaphragm; the infundibulum is compressed and distorted to the right of the tumor and enhances more than the tumor itself (the arrow in B identifies the stalk as it passes to the compressed pituitary in the floor of the sella). (C and D) T1-weighted, post-gadolinium coronal and sagittal images 6 weeks after the second transsphenoidal surgery, with complete resection of the suprasellar, intracranial component of tumor. The infundibulum is now more easily identified, although still deviated to the right (arrow in D). The normal pituitary gland has expanded to fill more of the sella (best appreciated on the sagittal view, C).

Three weeks later, she reported recrudescence of symptoms of hyperthyroidism (diaphoresis and palpitations), with recurrent evidence of biochemical hyperthyroidism and GH excess; methimazole and octreotide were re-introduced and 2 weeks later thyroid function normalized, T_4_ 10.2 (5.0–11.0 μg/dl) and TSH 4.9 (0.4–5.5 μU/ml). Residual adenomatous tissue was present on sellar imaging (Fig. 2A and B); the tumor appeared to have descended from the suprasellar space into the enlarged sella, sitting above and distorting the infundibulum and compressing the normal gland, both of which enhanced briskly. Repeat, extended transsphenoidal surgery was undertaken. At surgery, the residual tumor was located wholly above the diaphragm sella, which was intact; an extended, transsphenoidal, suprasellar approach, passing above the sella and pituitary and then approaching the tumor from above the diaphragm, was needed to identify and resect the tumor in the suprasellar space; the infundibulum was identified in the suprasellar, intracranial space, displaced to the right by the tumor, and then identified passing through the diaphragm, to the normal gland within the sella; it was left undisturbed (Fig. 2B). Methimazole and octreotide were discontinued again on the day of surgery. Repeat immunohistochemistry of the resected tissue was identical to the initial histological findings. Ki-67 was 2.1 and <5% of adenoma cells were p53 positive. Normalization of thyroid function and GH levels was observed post-operatively and has persisted, with complete resolution of the hyperthyroidism (Table 2). Post-operative MRI performed 6 weeks after surgery confirmed gross total resection of the intracranial component of the tumor; the infundibulum remained distorted to the right and is well visualized (Fig. 2C and D). At most recent follow-up, 3 years after the initial pituitary surgery, she has residual sleep apnea and continues to use ‘C-PAP’ nightly but is otherwise endocrinologically and neurologically normal.

**Table 2:** Serial thyroid function, GF1 and GH levels over time

**Test**	**Presentation**	**Day 1-post-op** (first TSS)	**Day 5-post-op** (second TSS)	**Follow-up** (7 months)	**Normal range**
T_4_ (μg/dl)	11.4	12.9	2.8	6.6	5–11
TSH (μU/ml)	2.8	3.7	0.04	1.2	0.4–5.5
T_3_ (ng/dl)	185	215	32	96	94–170
GH (ng/ml)	1.58	2.34	0.2	<0.05	0.01–0.97
IGF1 (ng/ml)	747	471	138	120	87–267

TSS, transsphenoidal surgery; T_4_, total thyroxine; TSH, thyroid-stimulating hormone; T_3_, tri-iodothyronine; GH, growth hormone; IGF1, insulin-like growth factor 1.

## Discussion

Plurihormonality does not always translate into the clinical features of hormonal excess. In addition to being distinct from one another with varying degrees of prominence, the clinical signs of hyperthyroidism and acromegaly can overlap. Patients with thyrotropic and somatotropic adenomas tend to present when the adenomas are large and frequently can be invasive and less amenable to surgical cure. Cure rates after surgery have been reported in around a third of patients with invasive tumors (5). Pre-operative somatostatin analog treatment can reduce tumor size and control thyroid and GH hypersecretion. Further options after surgery include radiotherapy and/or somatostatin analogs. Somatostatin analogs have been shown to result in euthyroidism in most cases of thyrotropinomas and ∼50% achieve a reduction in pituitary mass size; dopamine agonists have also been used, but with more limited success in thyrotropinomas (6) (7).

In our case, the predominant symptoms were those of hyperthyroidism, although the clinical signs of hypertension, cardiomyopathy, and sleep apnea could be a clinical manifestation of acromegaly or morbid obesity as well. Interestingly, immunohistochemistry with antibodies directed against TSH showed rare and sparse positive cells. In contrast, although IGF1 levels elevated to approximately three-times normal and showed diffuse robust staining against GH in the adenoma, the patient did not exhibit signs of acromegaly. One explanation is that the associated ‘classic’ signs often lag behind the elevation of IGF1/GH. Another plausible explanation would be that even though the pituitary macroadenoma has been present for some time as indicated by its size (8), production of excessive somatotropins from the adenoma could have been a recent phenomenon. Secretion of GH with low bioactivity from a ‘silent’ somatotrope adenoma could also explain the relative absence of the clinical signs of acromegaly (9). Not all cases of biochemical and clinical TSH/GH co-secreting pituitary adenomas display immunopositivity to TSH and/or GH. Negative immunostaining for TSH in co-secretory TSH/GH adenomas has been demonstrated, which could be due simply to low TSH levels in the adenoma (10). There appears to be a clinical and immunohistochemical spectrum present in plurihormonal tumors, and electron microscopy has been utilized by some to evaluate tumor cell lines in experimental animals (11). *De novo* transformation of a thyrotropinoma to a thyro-somatotrope adenoma appears to be exceedingly rare. The only case reported arose in a patient with hyperthyroidism from a thyrotropinoma, who developed evidence of GH co-secretion only after treatment with octreotide for 1 year. The authors speculated that a series of genetic mutations resulted in transformation of a monoclonal adenoma to a plurihormonal tumor (12). The PIT1 protein, which is a transcription factor whose presence is required *in utero *for normal development of the mammosomatropes that produce GH and PRL, is also required for the maintenance of normal expression of GH, prolactin, and TSH after the separate embryonic origin of thryotropes and has been postulated to play a role in the development of co-secretory pituitary adenomas (13). A recent study has demonstrated that the degree of β-TSH immunoreactivity appears to be associated with the degree of hyperthyroidism (14).

In summary, this case highlights an unusual patient with a rare TSH/GH co-secreting pituitary adenoma in whom symptomatic hyperthyroidism was the dominant and only clinical feature, without acromegaly. Immunohistochemistry was positive for GH but only rarely and sparsely positive for TSH. Secondly, while the patient had symptomatic relief of the signs and symptoms of hyperthyroidism for several weeks after what was thought to be gross total resection of a macroadenoma delimited by an elevated but anatomically intact diaphragm sella, the rapid recrudescence of hyperthyroidism led to early reappraisal and identification of residual component of tumor located wholly within the supra-diaphragmatic, intracranial space. Resection of the invasive, intracranial component of the adenoma led to resolution of biochemical evidence of acromegaly and the patient's clinical hyperthyroidism.

## Author contribution statement

All authors were involved in the preparation and writing of the manuscript.
